# Oxidative stress regulation and related metabolic pathways in epithelial–mesenchymal transition of breast cancer stem cells

**DOI:** 10.1186/s13287-023-03571-6

**Published:** 2023-11-28

**Authors:** Raheleh Farahzadi, Behnaz Valipour, Ezzatollah Fathi, Samaneh Pirmoradi, Ommoleila Molavi, Soheila Montazersaheb, Zohreh Sanaat

**Affiliations:** 1 Hematology and Oncology Research Center, Tabriz University of Medical Sciences, Tabriz, Iran; 2 Department of Anatomical Sciences, Sarab Faculty of Medical Sciences, Sarab, Iran; 3 Department of Clinical Sciences, Faculty of Veterinary Medicine, University of Tabriz, Tabriz, Iran; 4 Cellular and Molecular Research Center, Cellular and Molecular Medicine Research Institute, Urmia University of Medical Sciences, Urmia, Iran; 5 Department of Pharmaceutical Biotechnology, Faculty of Pharmacy, Tabriz University of Medical Sciences, Tabriz, Iran; 6 Molecular Medicine Research Center, Tabriz University of Medical Sciences, Tabriz, Iran

**Keywords:** Epithelial–mesenchymal transition, Oxidative stress, Reactive oxygen species, Metabolic pathways, Transcription factors, Signaling pathways

## Abstract

Epithelial–mesenchymal transition (EMT) is a cell remodeling process in which epithelial cells undergo a reversible phenotype switch via the loss of adhesion capacity and acquisition of mesenchymal characteristics. In other words, EMT activation can increase invasiveness and metastatic properties, and prevent the sensitivity of tumor cells to chemotherapeutics, as mesenchymal cells have a higher resistance to chemotherapy and immunotherapy. EMT is orchestrated by a complex and multifactorial network, often linked to episodic, transient, or partial events. A variety of factors have been implicated in EMT development. Based on this concept, multiple metabolic pathways and master transcription factors, such as Snail, Twist, and ZEB, can drive the EMT. Emerging evidence suggests that oxidative stress plays a significant role in EMT induction. One emerging theory is that reducing mitochondrial-derived reactive oxygen species production may contribute to EMT development. This review describes how metabolic pathways and transcription factors are linked to EMT induction and addresses the involvement of signaling pathways.

## Background

Breast cancer is the most prevalent malignancy among women worldwide. Despite great advancements in diagnostic and treatment approaches, metastasis and drug resistance still account for many breast cancer deaths [1]. According to the expression profile of estrogen receptor (ER), progesterone receptor (PR), and human epidermal growth factor receptor 2 (HER2), four major molecular subtypes were identified in breast cancer. These include luminal A (ER^+^, PR^+^), luminal B (ER/PR^+^, HER2^+^), HER2^+^, and basal-type breast cancer or triple-negative breast cancer (TNBC) with the absence of mentioned receptors [2].

Epithelial–mesenchymal transition (EMT) is a critical process by which polarized epithelial cells lose their adhesion capacity and are converted to motile mesenchymal stem cells (MSCs) [3, 4]. Indeed, a series of biological events are involved in the EMT program. EMT provides breast cancer cells (BCCs) with enhanced invasiveness and metastatic behavior and, importantly, endows stem cell-like properties [5]. EMT is strongly associated with the metabolic rewiring of glucose, amino acid, and lipids [6]. As a result of this metabolic reprogramming in BCCs, a series of events occurred, including stimulating the expression of EMT-related transcription factors (EMT-TFs), adoption of mesenchymal phenotype, and acquisition of breast cancer stem-like cell properties. These events lead to cancer stemness and metastatic-related behaviors [6, 7]. Growing evidence demonstrated that breast cancer stem cells (BCSCs) exhibit a plasticity transition between a proliferative state (epithelial-like phenotype) and a quiescent and invasive state (mesenchymal-like phenotype). This plasticity facilitates the tumorigenic behavior of BCSCs. Therefore, a better understanding of the underlying mechanisms for altered metabolism and its consequences during the EMT process may help to find novel possible targets for treating breast cancer [8].

Cancer stem cells (CSCs), also called tumor-initiating cells (TICs), are a small subpopulation of heterogeneous cells within tumor cells. CSCs are involved in cancer cell initiation, progression, and metastasis [9, 10]. Although CSCs comprise a small fraction of total tumor mass, they can spread and are likely to reactivate the quiescent state of cancer cells to the recurrent population [11]. Accumulating evidence revealed that CSCs are intrinsically resistant to anticancer therapy, as evident by their presence in tumor niches after chemotherapy or radiation therapy. Relying on this, CSCs represent a novel target in treating breast cancer [11].

Oxidative stress plays a pivotal role in a variety of anticancer strategies, such as radiation therapy. Oxidative stress can affect all cell systems through a higher concentration of reactive oxygen species (ROS). Tumors, in particular, CSCs exhibit a tight regulation of ROS levels, which is a necessary factor for resistance, cell signaling, and recurrence. In all cell compartments, various species of ROS are produced, and each has a remarkable impact on tumor progression, tumorigenesis, or therapy [12].

Like normal stem cells, CSCs possess lower intracellular ROS levels than tumor bulk. Lower amounts of ROS in CSCs maintain a stem cell-like phenotype and offer resistance to radiation or chemotherapy. This is partly related to less DNA damage occurring during therapy, leading to cancer recurrence after radiotherapy or chemotherapy [13]. Nevertheless, it is unclear how drug-resistant CSCs respond to oxidative stress [14]. Various outcomes are associated with increased ROS, including genetic instability, a hallmark of cancer cells. Besides, oxidative stress affects lipid metabolism by producing lipid peroxidation (LPO) products [15]. LPO and the formation of reactive aldehydes are significant end products that are considered essential biomarkers for various diseases such as cancer. 4-Hydroxy-2-nonenal (HNE) is a reactive aldehyde produced by cells during oxidative stress. HNE involves multiple signaling pathways and affects cellular events such as proliferation, differentiation, and apoptosis [16]. It has been found that nuclear factor erythroid 2-related factor 2/Kelch-like ECH-associated protein 1 (Nrf2/Keap1) can be affected by HNE. Indeed, Nrf2/Keap1 can alleviate oxidative stress by regulating the expression of Nrf2, as an antioxidant transcription factor. Indeed, Nrf2 plays a pivotal role in the defense against oxidative damage by activating a set of cytoprotective genes. Under the normal state, Nrf2 is inactive by binding to Keap1. However, upon binding HNE to the cysteine residue of Keap1, Nrf2 can escape from the Keap1-mediated repression. As a result, Nrf2 can migrate into the nucleus and activate antioxidant gene expression, enabling cells to survive against oxidative challenges [17, 18]. However, Li et al. showed that HNE promoted cell proliferation and angiogenesis in breast cancer by hypoxia-inducible factor (HIF)-1α stabilization [19].

This review aims to review the fundamental metabolic pathways involved in EMT development in breast cancer, focusing on oxidative stress and the metabolisms of amino acids, glucose, and lipids.

## EMT process in breast cancer

EMT is strongly associated with the enhanced tumor-initiating and metastatic potential of BCCs. EMT can increase invasion, mobility, and resistance to apoptotic stimuli and therapeutic regimens in cancer [20, 21]. EMT process is characterized by simultaneous downregulation of epithelial markers such as E-cadherin and upregulation of mesenchymal markers such as N-cadherin and vimentin [22, 23]. A set of TFs regulates the EMT program, including Snail, Twist-related protein (Twist), and zinc finger E-box binding homeobox (ZEB), whose differential expression leads to the EMT induction in breast cancer [24]. EMT-TFs also alter the expression and activity of downstream signaling cascades implicated in stemness phenotype, invasiveness, and metastasis, highlighting the oncogenic role of EMT-inducing TFs [25, 26]. In this regard, blocking the activation of EMT-inducing TFs is the most effective way to impede breast cancer's invasion and metastatic behavior [27].

As a result of the EMT process, BCCs acquire mesenchymal phenotype and stem cell-like properties, a necessary hallmark in breast cancer [28, 29]. In other words, EMT endows BCSC-like features to BCCs that lead to the acquisition of resistance to elimination by therapeutics [30]. Fischer et al. reported that BCCs undergoing EMT program could resist cyclophosphamide. This is possible due to damaged apoptotic tolerance and a higher level of chemoresistance genes [31].

Given the rapid proliferation of cancer cells, these cells alter their metabolic pathways to generate sufficient ATP and vital intermediates for their survival [32]. Under nutrient-deprived and hypoxic states, BCCs undergo EMT and metabolic rewiring to provide energy demand for motility and invasiveness [33, 34]. However, little is known about how EMT regulates this metabolic adaptation.

Collectively, EMT is regulated by multiple factors that include transforming growth factor-β (TGF-β), Wnt, Notch, and their related signaling proteins such as nuclear factor kappa B (NF-κB), phosphatidylinositol 3-kinase (PI3K)/Akt, and extracellular signal-regulated kinase (ERK). These signaling cascades are associated with tumor initiation and progression in response to various stressors, for instance, hypoxic status, metabolic or oncogenic stress, inflammatory conditions, and physical constraints. These signalings also activate EMT-inducing TFs, including Snail, ZEB1/2, and Twist1/2 [35].

## Metabolic pathways which induce the EMT process in breast cancer

### Amino acid metabolism

Accumulating reports indicate that amino acid metabolism is critical for maintaining cellular homeostasis [5]. Cancer cells need increased amino acid levels for rapid growth/proliferation. In addition to being used as substrates for biosynthetic purposes, amino acids act as metabolic regulators in supporting cancer cell growth [36]. Cancer cells display enhanced demand for nonessential amino acids and depend on exogenous sources or upregulated de novo synthesis [37]. Upregulation of enzymes associated with amino acid metabolism was found in breast cancer with high metastatic potentials. However, how altered amino acid metabolism modulates EMT development in breast cancer is unclear. Halldorsson et al. surveyed the metabolic profiling in the breast epithelial cell line (D492) and its EMT-derived cell line (D492M) with mesenchymal characteristics. They showed that glycolysis and oxidative phosphorylation were higher in the epithelial phenotype, while the mesenchymal phenotype relies more on fatty acid oxidation and amino acid anaplerosis. Amino acid metabolism plays a crucial role in the development of EMT [38]. With this notion, this part focuses on glutamine, asparagine, and cystine metabolism, which have partially been implicated in the EMT process.

Glutamine is the most abundant free amino acid in the human body, with numerous roles in biosynthetic and cellular processes. The marked increase in glutamine metabolism is a common metabolic alteration in many cancer types. Glutamine is highly consumed by most cancer cells and is considered the second cellular demand after glucose [39, 40]. Glutaminolysis is an essential process by which glutamine is converted to tricarboxylic acid cycle (TCA) metabolites (α-ketoglutarate) by a series of enzymes. The TCA cycle exerts its action by oxidative phosphorylation and energy production. Upon the entrance of glutamine into the cell, glutaminase (GLS) catalyzes convert of glutamine to glutamate and ammonia. GLS is expressed in two types in mammals: GLS1(kidney-type glutaminase) and GLS2 (liver-type glutaminase). GLS1 is abundantly expressed in many cancer types, and a high level of it is correlated with a poor prognosis. GLS2 function in cancer is not entirely understood. Some studies have reported that GLS2 overexpression reduces tumor growth, suggesting that GLS2 acts as a tumor suppressor.

Glutaminolysis is also required for metabolic reprogramming in cancer cells [41]. TGF-β increases the expression of GLS1 and enhances the intracellular catabolism of glutamine [42]. Dlx-2 is a transcription factor that plays an essential role in TGF-β and Wnt-induced EMT by Snail activation. This indicates the importance of Dlx-2 for the EMT program and the migration and invasion of cancer cells [43]. It is well established that silencing of GLS1 can prevent the EMT process. Indeed, GLS1 is the first enzyme in glutamine anaplerosis under the control of the MYC oncogene. GLS1 expression is enhanced in breast cancer and promotes tumor growth and metastasis [44].

Interestingly, GLS1 and GLS2 have different expression patterns in multiple cancers. GLS1 is highly expressed in some tumors due to direct regulation by oncogenes such as MYC and KRAS [45]. GLS2 is directly regulated by p53, p63, and p73 [46]. Consequently, EMT induction suppresses GLS2 expression and promotes glutamine independence even in conditions with low glucose and GLS presence. Ramirez-Peña et al. found that GLS2 re-expression could enhance glutamine consumption and decrease sphere formation. Given the important role of transcription factor Forkhead box C2 (FOXC2) in maintaining stem cell-like features and mesenchymal phenotype, inhibition of FOXC2 expression and subsequent EMT inhibition could restore GLS2 expression as well as glutamine utilization in cancer cells undergoing EMT. High expression of GLS2 in patients with breast cancer is inversely associated with the EMT program [39]. It can be inferred that high GLS2 expression exhibited less aggressive characteristics and improved survival in breast cancer patients [47]. Beyond feeding TCA, glutamate participates in the antioxidant defense system by glutathione synthesis (GSH), which neutralizes ROS. The TCA cycle provides carbon and nitrogen sources for synthesizing fatty acids, amino acids, nucleotides, and all intermediates necessary for GSH synthesis [48].

Asparagine is one of the essential nonessential amino acids in humans. Higher asparagine levels are strongly associated with EMT driving and subsequent metastatic behavior in BCCs. It has also been found that limiting asparagine bioavailability attenuates EMT-driving proteins. In this regard, increased activity of asparagine synthetase or asparagine intake results in higher metastatic potential and vice versa [49, 50]. Asparagine synthetase mediates the synthesis of asparagine from glutamine and aspartate. Therefore, targeting this enzyme reduces asparagine content and EMT program, alleviating the invasiveness and metastasis in breast cancer [51]. Limiting asparagine content through deamination by the L-asparaginase enzyme reduces EMT driving and breast cancer metastasis [52].

Another amino acid that contributes to the EMT program is cystines. Cystine is formed by oxidizing two cysteine molecules with a disulfide bond formation. It can be reversibly converted to cysteine by a reducing agent. Indeed, cysteine is the predominant circulating form of cysteine [53]. Cystine addiction is a crucial metabolic hallmark in cancer, especially in TNBC.

In contrast, luminal BCCs are cystine-independent. This addiction may be related to EMT-driving proteins [54]. It was reported that basal-type BCCs displayed rapid programmed necrosis via cystine deprivation. Conversely, little death was observed during cystine deprivation in the case of luminal-type BCCs (cystine-independent type). It has also been reported that overexpression of miR-200c inhibited EMT induction in cystine-addicted MDA-MB-231 cells [55]. With this notion, it can be inferred that cystine plays a crucial role during EMT in BCCs, but it is not clear how it affects EMT [56].

### Glucose metabolism

Under aerobic conditions, normal cells obtain their energy through cytosol glycolysis, followed by mitochondria oxidative phosphorylation. In contrast, cancer cells prefer to obtain energy from glycolysis in the cytosol even in the abundant oxygen supply, a phenomenon known as the “Warburg effect” [57]. This can be attributed to the fact that the yield rate of glycolysis is much faster than oxidative phosphorylation, even though energy production is much lower via the glycolytic pathway. In this context, aerobic glycolysis meets nutrient demands for cancer cells' fast proliferation and growth. Such metabolic reprogramming of glucose has been observed in many cancer types. The increasing glycolytic rate can provide glycolysis-related intermediates for biomass biosynthesis in cancer cells [58, 59].

In breast cancer, EMT is accompanied by increased aerobic glycolysis and upregulation of glycolysis-associated enzymes [60]. In the glycolytic pathway, pyruvate kinase catalyzes the last step of glycolysis by transferring phosphate groups from phosphoenolpyruvate to ADP, producing ATP and pyruvate. Depending on tissue types, four isoforms of pyruvate kinase exist, including L, R, M1, and M2. Of note, pyruvate Kinase M2 (PKM2) is exclusively expressed in many cancers, such as breast cancer. PKM2 enhances the Warburg effect and tumor growth and stimulates the expression of HIF-1α [61]. A recent study by Li et al. showed that PKM2 knockdown in gastric carcinoma restrained invasion and migration in cancer cells by inhibiting EMT induction. This is mediated by inhibiting E-cadherin and promoting the expression of mesenchymal proteins, including N-cadherin and vimentin. Besides, PKM2 knockdown suppressed the HIF-1α in cancer cells [62]. The EMT process is orchestrated after the transportation of PKM2 into the nucleus and the repressing of E-cadherin expression. In the nucleus, PKM2 interacts with TGF-β-induced factor homeobox-2 (TGIF2) and localizes histone deacetylase 3 (HDAC3) in the promoter of the CDH1 gene, repressing its expression. Indeed, the CDH1 gene encodes E-cadherin, an epithelial cell adhesion molecule. A higher level of HDAC3 is correlated with poor prognosis in multiple cancers. Thus, suppression of E-cadherin can lead to the loss of EMT induction [63, 64]. This is the non-canonical activity of PKM2, independent of the classic function of this enzyme (catalysis of glycolysis). These considerations also suggest that metabolism-associated enzymes exhibit alternative roles in cancer cells that may be crucial for the survival of cancer cells [65].

Several lines of evidence declared overexpression of PKM2 in many types of cancers, such as pancreatic ductal adenocarcinoma [66], ovarian cancer [67], and gastric cancer [68], that favor the glycolytic pathway to achieve nutrients demand much faster than oxidative phosphorylation. Considering the upregulation of PKM2 in most cancer cells, it can serve as a potential therapeutic target for cancer therapy.

Pyruvate dehydrogenase kinase (PKD) is another fundamental enzyme in glucose metabolism composed of four isoenzymes, including PDK1 (responsible for the Warburg effect in cancerous cells), PDK2 (with abundant and constitutive expression in all tissues), PDK3 (low expression in tissues), and PDK4 (the most attractive kinase among isoenzymes). PDK1 exerts its effect by phosphorylation and inactivating the pyruvate dehydrogenase complex [69, 70]. The pyruvate dehydrogenase complex maintains glucose homeostasis in mammals by entering carbohydrates (via pyruvate) into the TCA cycle [71]. It has been shown that PDK1 is required for liver metastasis in breast cancer patients via the promotion of glycolytic metabolism [72]. PDK1 is also necessary for EMT induction via downregulating mesenchymal markers. Du et al. reported that specific deletion of PDK1 in BCCs hindered tumor initiation, progression, and metastasis in a mouse model. Indeed, deficiency of PDK1 expression inhibited the EMT-induced process [73].

The most crucial glycolytic enzyme is PDK4 which phosphorylates the pyruvate dehydrogenase complex and inactive it, thereby preventing pyruvate entrance into the TCA cycle. Upon inhibition of pyruvate conversion to acetyl-CoA, a series of events occurred: reducing the metabolite flux into the TCA cycle, suppressing aerobic respiration, and switching to the glycolytic pathway [74]. Multiple reports confirmed the oncogenic role of PDK4 in human cancers such as colorectal cancer [75] and bladder cancer [76]. Indeed, elevated levels of this enzyme are highly associated with aggressiveness and chemoresistance in cancer cells [70]. Taken together, the inhibition of PDK can be a therapeutic strategy for cancer treatment.

Phosphoglucose isomerase (PGI), also called phosphohexose isomerase, is a glycolytic enzyme that mediates the interconversion of glucose 6-phosphate and fructose 6- phosphate in a reversible manner. PGI affects EMT during the early stage of cancer metastasis and mesenchymal–epithelial transition (MET) at the final stage of cancer metastasis in breast cancer [77]. EMT occurs during an early stage of cancer progression, while MET is a crucial step for metastasis and allowing colonization to secondary sites. The autocrine motility factor (AMF) is secreted by tumor cells and is abundant at tumor sites. It was reported that PGI/AMF overexpression led to EMT in normal human breast epithelial cells, resulting in cells escaping from the primary tumor. In addition, inhibiting PGI/AMF expression triggered MET in the aggressive form of BCCs, allowing their colonization and development at secondary sites. MET is considered a central step for metastasis at a late stage. Molecular analysis also revealed that PGI/AMF could inhibit the expression of epithelial markers and enhance the expression of mesenchymal markers [77].

Enolase 1 (ENO1) is a metalloenzyme that mediates the conversion of 2-phosphoglyceric acid (2-PGA) to phosphoenolpyruvic acid (PEP), a critical step in the glycolytic pathway. Expression of ENO1 was found to be upregulated in numerous cancers [77], for instance, head and neck [78], lung [79], and so on. In line with this, Zhou et al. found that lung adenocarcinomas display an increased level of ENO1 expression. According to their results, silencing ENO1 expression retarded the glycolytic pathway, inhibited the EMT program, and induced apoptosis [80]. Based on proteomic analysis of gastric cancer cells, ENO1 is an essential component of a protein–protein interaction network involved in tumor growth and metastasis; thus, silencing of ENO1 led to cell cycle arrest and growth inhibition of gastric cancer cells [81].

**Fig. 1:**
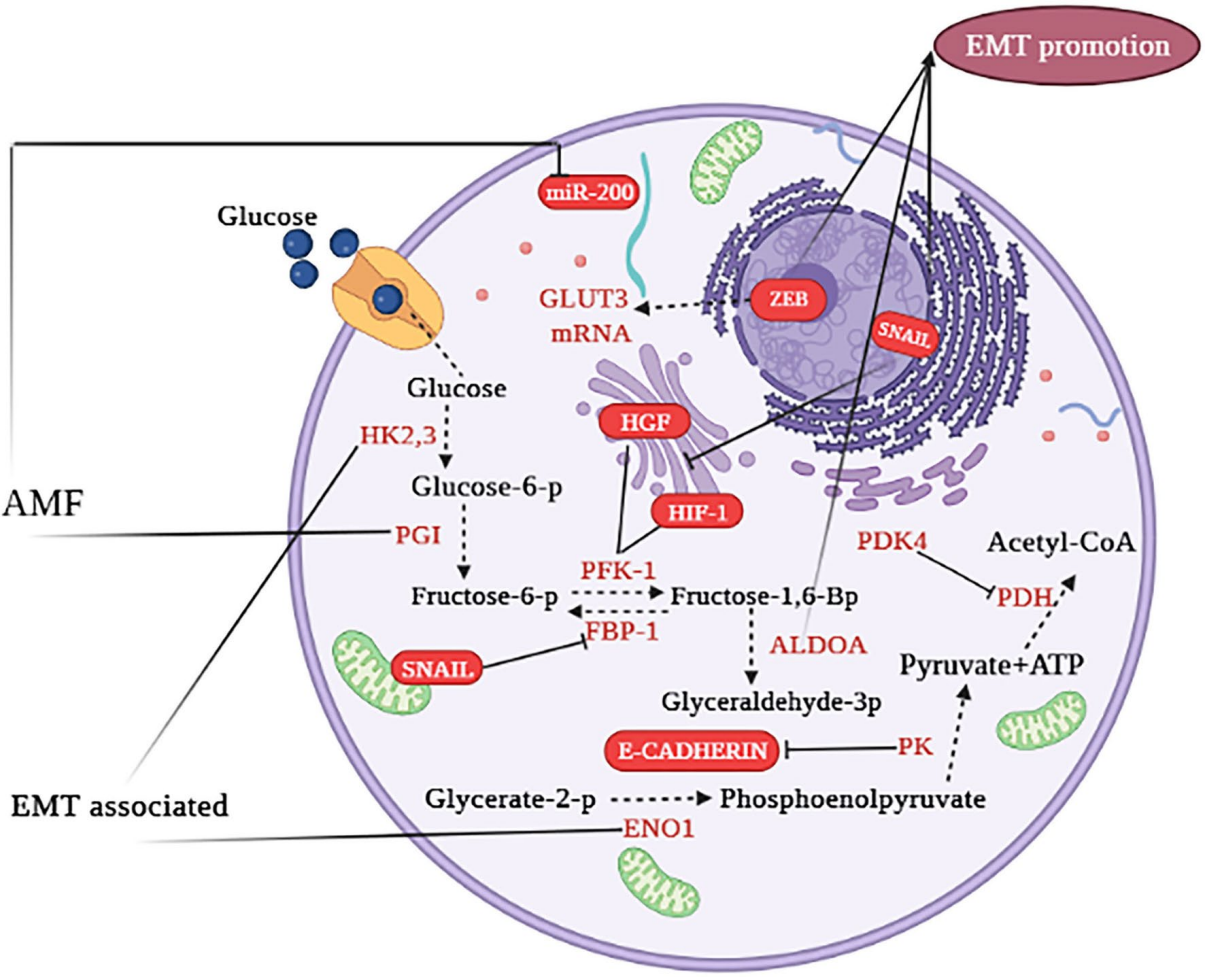
Metabolic cross talk of glycolytic pathways in the EMT program. The yellow font represents enzymes, red font represents EMT-related factors. → indicates induction; ⊣ indicates inhibition. Intermediate reactions are highlighted in red dots (not shown) (illustration created by biorender.com). This figure adapted from Fig. 1 of reference number 5 [5]

Glucose transporters (GLUT) are essential mediators in glucose metabolism, by which glucose shuttles across the plasma membrane. Of note, this is the first step of the glycolytic pathway. These transporters are membrane-embedded proteins in all cell types that facilitate the entry of glucose (the primary fuel of most cells) from the surrounding area into cells. GLUT1 and GLUT3 shuttle glucose in an insulin-independent manner. GLUT1 is constitutively expressed in multiple organs with a high expression in the fetus, whereas GLUT3 is specifically expressed in neurons. In other words, GLUT3 is the most abundant transporter in neurons [82]. In the case of cancer cells, an increased expression of GLUT1 and GLUT3 was detected, facilitating glucose uptake with independence from insulin levels. Notably, high levels of GLUT1 or GLUT3 usually correlate with poor outcomes in cancer patients [83–85]. In laryngeal carcinoma, the expression of GLUT1 is associated with EMT-related markers such as vimentin and N-cadherin [86]. In human non-small cell lung cancer cell lines, GLUT3 is overexpressed in mesenchymal cells, not epithelial cells. The binding of transcription factor ZEB1 to the GLUT3 gene induces transcription of this transporter, indicating the critical role of GLUT3 in EMT development. Inhibiting the expression of GLUT3 caused a decline in glucose import. GLUT3 is induced during the EMT and promotes tumor cell proliferation in non-small cell lung cancer [87]. In Fig. 1, we briefly summarize the role of the glycolytic pathway in the EMT process [5].

Hypoxia is an inducer in switching from oxidative phosphorylation into glycolysis during the rapid proliferation of cells. Breast tumors display decreased oxidative phosphorylation due to a high proliferation rate [88]. Mutations in mitochondrial DNA (mtDNA) or low mtDNA content may result in decreased oxidative phosphorylation activity [89]. Consistently, Guha et al. demonstrated that reducing mtDNA content could activate the calcineurin-dependent mitochondrial retrograde pathway. This resulted in EMT-like reprogramming to fibroblastic phenotype in human mammary epithelial cells.

Interestingly, the reduction of mtDNA induced EMT and generated BCSCs in human mammary epithelial cells. Of course, these changes can be reversed by restoring the mtDNA content. A reduced mtDNA content and decreased oxidative phosphorylation activity could offer novel targets for treating metastatic cancer [90].

### Lipid metabolism

Aside contribution of glucose metabolism in cancer cells, aberrant lipid metabolism can develop EMT in breast cancer, even though it gained less attention compared to aerobic glycolysis. De novo lipogenesis synthesizes fatty acids from non-lipid sources, such as excess carbohydrates, as acetyl-CoA. Fatty acids are needed to synthesize biological membranes, signaling molecules, and energy supply. Fatty acids can be esterified to glycerol, forming triglycerides [91].

Lipid biogenesis is generally increased in cancer cells to meet all lipids requirements for synthesizing membranes and signaling molecules. Besides, cancer cells accumulate lipids in the form of droplets more than normal cells. Given the elevated level of lipogenesis in cancer cells, it can be a target for cancer treatment [92]; however, little is known about the interplay between lipids and the EMT process.

Fatty acid synthase (FASN) catalyzes de novo lipogenesis. This enzyme plays a critical role in the development of EMT in breast cancer. In other words, FASN promotes breast cancer metastasis by altering lipid metabolism. Accordingly, FASN can be considered a therapeutic target in breast cancer treatment [93]. As reported by Li et al., cerulenin, as a FASN inhibitor, inhibited the viability, migration, and EMT in BCCs [94]. It was evident that expression of FASN is commensurate with the tumor's grade and resistance to therapeutic agents such as tamoxifen (estrogen receptor antagonists), as observed in endocrine therapy in ER^+^ breast cancer. Using FASN inhibitor remarkably impeded the proliferation rate in tamoxifen-resistant cells compared to the parental cells [95]. Another similar study by Menendez et al. provided evidence regarding the FASN role. They revealed that the FASN inhibitor prevented the estrogen tumor-promoting effects of tamoxifen and completely restored the tamoxifen sensitivity in resistant BCCs overexpressing ER^+^/HER2 receptors [96]. Despite that effects, FASN silencing can induce TGF-β1-induced EMT and metastasis [97].

Another critical lipogenic enzyme is acetyl-CoA carboxylase1 (ACC1), which is involved in the EMT in breast cancer. ACC1 catalyzes de novo lipogenesis by carboxylation of acetyl-CoA and the formation of malonyl-CoA, which is an intermediary metabolite with a signaling function. Besides, malonyl-CoA is associated with protein acetylation [98]. Garcia et al. provided evidence that ACC1 is the main contributor to breast cancer metastasis. They inhibit ACC1 as a point of convergence for EMT and invasion-inducing pathways, like leptin and TGFβ, which are present in obese patients with breast cancer. Leptin and TGFβ suppress ACC1 activity in breast tumor cells via phosphorylation by transforming growth factor-activated kinase 1 (TAK1). Upon suppressing ACC1 activity, tumor cells enhanced acetyl-CoA levels, protein acetylation, migration, and invasion, which is mediated by EMT and Smad2 acetylation. Relying on these findings, activating leptin or the TGFβ-induced axis prevented ACC1 expression and the EMT program. Accordingly, targeting the ACC1-mediated EMT may offer an attractive treatment strategy for breast cancer patients with obesity [99].

Growing evidence proposes that lipid-associated metabolic enzymes may be utilized as therapeutic targets to inhibit the EMT process in breast cancer. Given the aggressiveness of basal-like breast cancer/TNBC in terms of distant metastasis, high grade, and poor prognosis, the development of effective interventions is in demand. Wu et al. showed that overexpression of aldo–keto reductase 1 member B1 (AKR1B1) correlates highly with basal-like breast cancer. Mechanistic investigation revealed that Twist2 transcriptionally stimulates AKR1B1 expression and activates the NF-κB pathway. NF-κB can upregulate Twist2 expression, thereby forming a positive feedback loop. These events drive to EMT process and promote CSC-like properties in basal-type cancer. Expression of AKR1B1 enhances tumorigenicity and metastasis, while its knockdown acts inversely. Epalrestat is an inhibitor of AKR1B1 that can drastically suppress the EMT network. Accordingly, AKR1B1 may be a potential target for patients with TNBC [100]. Furthermore, it has been identified that the lipid transfer protein Nir2 promoted EMT progression and facilitated metastatic potential in breast cancer.

Indeed, overexpression of Nir2 led to reduced epithelial markers with a concomitant increase in mesenchymal markers. In contrast, suppression of Nir2 expression exhibited opposite effects. Nir2 exerts its impact by PI3K/AKT and the ERK1/2 pathways. Nir2 silencing prevented TGF-β1-induced EMT, indicating that Nir can be a valuable therapeutic target in breast cancer [101].

**Fig. 2:**
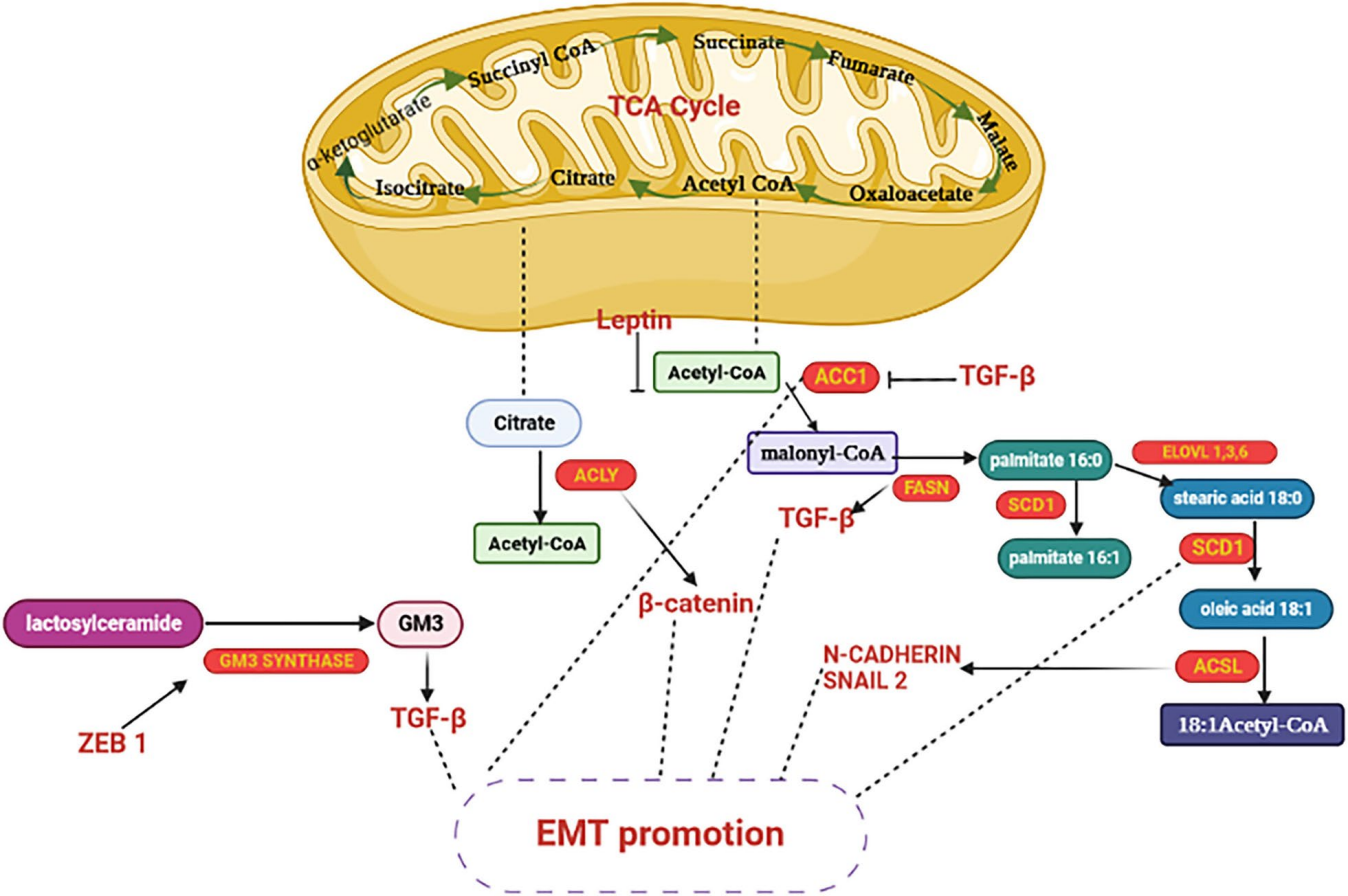
Interplay between fatty acid metabolism and EMT-related factors. Enzymes are identified in yellow font, whereas EMT-related factors are denoted in red font. → indicates induction; ⊣ indicates inhibition (illustration created by biorender.com). This figure adapted from Fig. 3 of reference number 5 [5]

As discussed earlier, BCSCs are a small fraction of primary tumors, and targeting BCSCs is a practical approach for preventing metastatic potential and, importantly, sensitizing cancer cells to chemotherapeutic agents. The ganglioside GD2 is upregulated in primary TNBC tumors compared to normal breast tissue. GD3 synthase (GD3S) is the regulatory enzyme for the biosynthesis of GD3 and GD2. It enhanced breast cancer metastasis by regulating EMT development. EMT-inducing signals elevated GD2 content as well as GD3S expression in BCCs. As a result, suppression of GD3S expression may provide novel insights into overcoming EMT in breast cancer [102]. Figure 2 summarizes the main lipid metabolism pathways interacting with the EMT process [5].

## The impact of ROS on EMT induction on BCSCs

ROS is a collective term for oxygen species with more reactivity than free oxygen. ROS can be divided into free radical ROS such as superoxide anion (·O_2_^−^), nitric oxide (·NO), and hydroxyl ion (·OH), as well as non-radical ROS, including hydrogen peroxide (H_2_O_2_) and peroxynitrite (–ONOO). H_2_O_2_ and O_2_^−^ are well-characterized ROS. Under physiological conditions, ROS is produced as byproducts in the mitochondria from the electron transport chain. ROS can also be generated from NADPH oxidases (NOXs) and peroxidases in cellular organelles and compartments such as peroxisomes, endoplasmic reticulum, and cell membranes [103]. ROS concentrations are increased in many cancer cells partly due to their higher metabolic activity [104]. Cancer cells produce ROS in the mitochondria via NOX as a ROS-generating enzyme. ROS has dual functions, including signaling molecules at lower concentrations and deleterious effects at above-threshold levels. The exact signaling pathway by which ROS can induce EMT is not clear. ROS contribute to the metabolic reprogramming of cancer cells and their surrounding microenvironment to facilitate tumor progression. ROS can also affect cellular signaling to drive the cancer phenotype by inducing EMT-associated pathways. NAD (P)H: quinone oxidoreductase-1 (NQO1) is an antioxidant enzyme. Upregulation and downregulation of NQO1 are closely correlated with reduced and increased susceptibilities toward oxidative stress, respectively [105]. Yang et al. reported that NQO1 overexpression in BCCs promoted glucose metabolism and metastatic potential. NQO1 could bind to liver-type pyruvate kinase (PKLR), which activates the AMP-activated protein kinase (AMPK) and AKT/mTOR signaling pathway, and ultimately induce glycolytic reprogramming and EMT in breast cancer. Indeed, NQO1 promoted glycolysis by supplying NADPH homeostasis. NQO1 suppression remarkably enhanced intracellular ROS and prevented EMT induction. Considering the supportive contribution of the NQO1/PKLR network in EMT progression, this may serve as an effective target for inhibiting the EMT process [106].

EMT-inducing ROS can be endogenously generated from the activation of ROS-producing enzymes, general oxidative stress, or by stromal cells within tumor niche. Indeed, the emerging mechanism paradigm by which ROS induces EMT is not absolute but strongly depends on the cellular context and tissue types [107]. Cichon et al. reported that ROS-mediated EMT processes in breast cancer progression. Matrix metalloproteinases (MMPs) have been recognized as specific inducers of EMT in breast cancer models. MMP-3 can induce EMT and malignant transformation by a pathway depending on ROS. The precise mechanism of how MMP-3-induced ROS stimulates EMT is still unknown. MMP-3 can increase Rac1b expression. They found that MMP-3/Rac1b-induced ROS could promote EMT in the breast cancer model. MMP-3-induced EMT was found to be dependent upon increased expression of Snail (the critical EMT transcription factor). The overexpression of Snail is due to the binding of p65 and cRel (subunits NF-κB) to the promoter region of Snail. This binding is dependent upon the activation of p65/cRel heterodimers by MMP-3-induced ROS [108]. In another report by Radisky et al., MMP-3 stimulated the EMT process by upregulating Rac1b in mammary epithelial cells. Indeed, EMT induction relies upon cellular ROS production [109]. Lee et al. reported that ROS-induced EMT is mediated by distal-less homeobox-2 (Dlx-2) /Snail signaling cascades in MCF-7 cells. Dlx-2 is one of the distal-less homeobox (Dlx) genes that act as an upstream mediator of ROS-mediated Snail expression. They also reported that ROS-induced EMT by the Snail activation represses the expression of E-cadherin in breast cancer cell lines [110].

Oxidative stress has a vital role in the pathophysiology of cancer. Indeed, normal and cancer cells maintain the balance/equilibrium between the generation and depletion of ROS [111]. Due to the high-rate metabolic activity in cancerous cells, the production of ROS is increased in mitochondria and endoplasmic reticulum. Based on the literature review, higher levels of ROS are associated with tumorigenesis. As mentioned above, ROS stimulates EMT induction, an essential step in metastasis initiation; however, the precise mechanism of ROS-induced EMT remains unclear. Given the significance of redox equilibrium in cancer cells, chemotherapy or radiotherapy targeting redox balance can eradicate most cancer cells [112].

Nevertheless, the unique redox system in CSCs and its underlying molecular mechanisms in protecting CSCs from ROS-induced cell death have not been completely understood [14]. Due to the plasticity and heterogeneity of CSCs, these cells exhibit diverse metabolic and redox states across different cancer types. There is a disparity in generated ROS in CSCs vs. non-CSC. Indeed, CSCs have specific metabolic demands. CSCs change their phenotype during metastasis via a transition from epithelial to mesenchymal-like states. CSCs attain invasive properties during the EMT proces**s** while leaving the initial tumor site. It is well established that ROS amounts and related metabolic activities differ between the epithelial and mesenchymal phenotypes of CSCs [113].

In CSCs, ROS occurs at decreased levels compared with non-stem cancer cells in some human breast cancers. CSC-enriched tumors experience less DNA damage than non-stem cancer cells upon exposure to radiotherapy. Indeed, overexpression of genes responsible for ROS defenses is observed among the CSC population with low levels of ROS. Such adaptation can reduce ROS production and protect CSCs from ROS’s detrimental effects. Accordingly, oxidative stress is one of the most critical mechanisms in regulating CSC traits [114].

Schieber et al. showed that epigenetic silencing of fructose-1,6-biphosphatase (FPB1) reduces ROS levels and promotes CSC populations and EMT phenotype in basal-like breast cancer. This can be due to the increased glycolytic flux following FBP1 silencing that leads to reduced levels of ROS. Two mechanisms were involved in this event: reduced mitochondrial respiration and elevated NADPH synthesis by pentose phosphate metabolism. Lower ROS levels can promote the EMT process and CSC phenotype in basal-like breast cancer. An important outcome of having a low level of ROS is the maintenance of a CSC subpopulation within breast tumors [115].

As discussed earlier, one of the consequences of oxidative stress is lipid peroxidation, which can affect various signaling pathways through reactive aldehydes, such as HNE, as a biomarker of oxidative stress. In this regard, Gašparović et al. investigated whether chronic oxidative stress and HNE-modified collagen of the microenvironment affect the EMT markers, antioxidant system, and the frequency of BCSCs. EMT is a critical event in cancer progression due to the conversion ability between differentiated epithelial cells and migratory MSCs. According to their findings, oxidative changes and particularly chronic oxidative stress caused changes in the proliferation and growth of BCSCs. Additionally, any EMT-related alteration can increase GSH and Nrf2 levels in BCSCs grown under chronic oxidative stress together with HNE-pretreated collagen. Chronic oxidative stress can act as a bidirectional regulator of BCSC fate. Low levels of HNE can enhance differentiation markers in BCSCs, whereas a higher level of HNE increases GSH, Nrf2, and specific EMT markers, thereby increasing resistance to therapeutic interventions [116].

Since BCSCs generate a lower amount of ROS than the corresponding tumor cells, breast cancer stem-like cells exhibit radioresistance, resulting in recurrence and distant metastasis after radiation therapy. ROS can act as an ionizing agent of radiation-induced death; accordingly, CSCs displayed less DNA damage compared to tumor cells. It seems that the overexpression of ROS-scavenging molecules contributes to these events. In other words, CSCs have an increased antioxidant capacity to sustain cellular ROS for CSC survival and resistance [117]. BCSCs have specific mechanisms for protecting cells from the genotoxic effects of ROS. In this regard, all genes encoding antioxidative enzymes are significantly upregulated in BCSCs. Besides, higher scavenging of ROS and lower production of it are other protective mechanisms reported in BCSCs. The latter phenomenon is attributed to the slow division of BCSC, which produces less ROS than regular cancer cells [118].

As evidenced, oxidative stress plays a critical role in EMT induction. It has been reported that reducing mitochondrial-derived ROS may contribute to EMT induction. Although BCSCs upregulate glycolysis-related genes, the response of mesenchymal and epithelial states of BCSCs to oxidant stress is mediated by diverse metabolic pathways and redox status. This plasticity enables BCSCs to transit between an epithelial-like state (with a high expression of aldehyde dehydrogenase) and a mesenchymal-like state (with the expression of CD24^−^CD44^+^). Importantly, elevated ROS levels can convert mesenchymal BCSCs to epithelial BCSCs. Therefore, mesenchymal BCSCs exhibit declined oxidative phosphorylation and ROS levels [54, 119].

CSCs, similar to normal stem cells, have decreased levels of ROS, which is an essential factor for stem cell maintenance, highlighting the presence of highly expressed ROS-scavenging molecules [120]. As mentioned above, Nrf2 is a key regulator of the antioxidant defense system that is increased in various models of CSCs. Nrf2 acts as an upstream regulator of NQO1 and contributes to the maintenance of CSCs properties, including quiescence, self-renewal, survival, and stress resistance [121].

C-terminal binding protein (CtBP) is a transcriptional co-repressor that affects various cellular processes. CtBP acts in an NADH-dependent manner and drives the EMT process in BCCs. Indeed, the expression of CtBP stimulates mesenchymal phenotype or/and BCSC features [122]. In contrast, CtBP depletion reverses the process and enhances DNA repair. Accordingly, CtBP downregulation significantly inhibits the EMT progression; thus, CtBP can be regarded as a pharmacologic intervention for suspending the EMT process. On the other hand, some conflicting views declare that the higher ROS level may contribute to EMT and BCSC-like features in breast cancer [123]. Further investigation is needed to elucidate the precise function of ROS in inducing the EMT process in breast cancer and BCSCs.

## ROS-dependent signaling pathways in CSCs

Normal stem cells and CSCs can scavenge ROS by utilizing multiple signaling pathways and transcriptional activities according to the following description:

### PTEN/PI3K/AKT/mTOR

The PI3K pathway is the most frequently activated in human cancers, implicating in cancer pathogenesis. Research has shown that the PI3K pathway plays a pivotal role in cell proliferation and survival of cancer cells [124]. As a result of activated PI3K/AKT/mTOR signaling, cell metabolism and glycolysis increase which in turn influences intracellular ROS levels as well as tumor development [125].

It is well known that phosphatase and tensin homolog deleted on chromosome 10 (PTEN) negatively regulates PI3K, acting as a direct antagonist. PTEN as a tumor suppressor encodes a protein with phosphatase function against proteins and phospholipids. The lipid phosphatase mediates the conversion of phosphatidylinositol 3,4,5-trisphosphate (PIP3) to phosphatidylinositol 4,5-bisphosphate (PIP2). PIP3 has been shown to be necessary for activating multiple downstream pathways, such as AKT [126]. Mutations in PTEN can cause PIP3 accumulation, resulting in the overactivation of the AKT pathway. It is worth noting that mutations or deletions of PTEN are involved in developing various types of cancers [127].

It has been identified that PI3K/AKT pathway is overactivated in CSCs. CSCs are the initiators of tumor neovascularization and are associated with tumor growth and invasion. It is evident that CSCs secrete proangiogenic factors. Besides, the transdifferentiation potentials of CSCs into vascular mural cells lead to the formation of non-endothelium-lined blood vessels [128]. As reported, PI3K/AKT activation could stimulate the production of vascular endothelial growth factor (VEGF) in CD133^+^ cancer stem-like cells in glioma. As a result, VEGF can induce both angiogenesis and vasculogenesis through the transdifferentiation of CSCs [129]. In support of this, another study found that the PI3K/Akt/mTOR pathway played a prominent role in maintaining breast cancer stem-like cells [130].

Sato et al. reported that ROS-induced p38 MAPK activation has a crucial role in controlling the differentiation, self-renewal, and tumor-initiating potential of glioma-initiating cells (GICs) derived from glioblastomas. According to their results, hydrogen peroxide solution at a dose of 100 μM could induce AKT phosphorylation and activate it in GICs. Accordingly, it can be inferred that the ROS-p38 axis controls the stemness of GICs [131].

In CSCs, a ROS-mediated oxidative environment is critical in regulating the catalytic activity of PTEN. The catalytic domain of PTEN has cysteines at various positions. Exposure of PTEN to H_2_O_2_ led to the inactivation of PTEN by forming a disulfide bond between cysteine 124 and cysteine 71. Thioredoxin is a critical mediator in redox signaling that reduces the PTEN disulfide bond and reactivates its function [132].

### Wnt pathways

The importance of the Wnt pathway is well established in regulating cellular behaviors. Beyond its early roles in embryonic development, Wnt controls cell proliferation, differentiation, migration, and polarity [133]. It has been proposed that CSCs of esophageal cancer are resistant to radiotherapy. COX-2 plays a crucial role in the radioresistance of esophageal cancer; thus, inhibiting COX-2 can be an effective strategy for radiosensitization. In this regard, Che et al. conducted a study to elucidate the CSC characteristics of radioresistant esophageal cancer cells and investigate the radiosensitization effect of a selective COX-2 inhibitor (NS398). According to their findings, radioresistant cells of breast cancer displayed CSC-like traits. Besides, β-catenin as the stem cell marker elevated in radioresistant BCCs. The NS398 could increase the radiosensitivity of resistant cells, possibly mediated by downregulating β-catenin expression [134].

Accumulating evidence declares that high ROS levels hindered β-catenin activation [135]. Nucleoredoxin is a member of the thioredoxin family involved in cell growth and differentiation. Nucleoredoxin as a redox regulator can interact with the Disheveled protein, affecting Wnt signaling. Overexpression of nucleoredoxin could selectively suppress the Wnt–β-catenin signaling pathway. Similarly, H_2_O_2_ hampered the association between disheveled with nucleoredoxin, inhibiting Wnt-β-catenin signaling [136].

Fructose-1,6-bisphosphatase (FBP1) is a rate-limiting enzyme in gluconeogenesis. FBP1 expression enhances ROS production. Elevated ROS levels can shift the interaction of β-catenin from transcription factor 4 (TCF4) to forkhead box O3 (FOXO3a) and thus prevents tumorigenicity in vitro and tumor formation in vivo. In other words, overexpression of FBP1 could increase oxidative phosphorylation and ROS generation and decline β-catenin activity by dissociation from TCF4. In basal-like breast cancer, loss of FBP1 increases CSC-like properties and tumorigenicity by β-catenin activation. Further investigation is needed to determine whether Wnt signaling directly affects this metabolic regulation [137].

### STAT Pathway

Signal transducer and activator of transcription 3 (STAT3) is the most investigated transcription factor in the Janus kinase signaling pathway (JAK)/STAT). It is overexpressed in tumor tissues and is positively associated with poor outcomes in cancer patients [138]. Hyperactivation of STAT3 is well documented in multiple cancers, including hepatocellular carcinoma [139], multiple myeloma [140], leukemia [141], breast cancer [142], and so on. In other words, aberrant activation of STAT3 is involved in malignant phenotypes of cancers. As an oncogene, STAT3 contributes to numerous cellular processes, including cell proliferation, differentiation, angiogenesis, survival, invasion, metastasis, immune response, and suppression of apoptosis in cancer cells. Given the self-renewal potential of CSCs to generate diverse cancer cells and tumor heterogeneity, inhibiting STAT3 can be an attractive strategy in tumor therapy [143, 144].

STAT3 activation by ROS could upregulate the self-renewal potential in prostate CSCs [145]. Additionally, elevated levels of aldehyde dehydrogenase (ALDH) in endometrial cancer can promote CSC traits through IL-6/JAK1/STAT3-mediate pathways, thereby blocking these pathways declined tumor growth and progression [146]. ALDH refers to multiple enzymes that oxidize the genotoxic aldehyde and is utilized as a CSCs marker for various cancers [147].

Cucurbitacin B, as a STAT3 inhibitor, can effectively inhibit gastric cancer progression by preventing STAT3 activity [148]. Similarly, Cucurbitacin B prevented lung cancer cell proliferation and promoted apoptosis by suppressing the IL-6/STAT3 pathway [149]. In the case of breast cancer, STAT3 is redox-sensitive, as H_2_O_2_ can decrease STAT3 binding to the serum-inducible factors, inhibiting the proliferation of cancer cells [150]. Notably, the STAT3 activity can be positively modulated by mTOR in breast cancer stem-like cells. On the other hand, it was shown that the PTEN could negatively regulate STAT3 and mTOR [151].

### Notch pathway

The Notch pathway is key signal transduction in the developmental stage by regulating cell fate and tissue formation. Current evidence proves that the Notch pathway has a major role in breast tumor progression. The Notch pathway is also essential for governing CSCs behavior [152]. BCSCs are known as the seeds of cancer development with indefinite proliferative potential. Notch and Wnt pathways are considered master developmental cascades, having vital roles in the development of CSCs and stemness maintenance. Moreover, the hypoxic microenvironment is involved in the maintenance of the stemness phenotype in BCSCs that is mediated by the activation of hypoxia-inducible factors (HIFs). HIF-2α activates the Notch and Wnt pathways to promote tumorigenicity and resistance of BCSCs [153].

Qiang et al. demonstrated that HIF-1α-mediated Notch signaling activation is critical for glioblastoma stem cell maintenance. Indeed, the Notch pathway has a fundamental role in regulating the ROS level in CSCs. This is mediated by PI3K/AKT pathway. In addition, AKT is overexpressed by Notch-mediated signaling in glioma stem cells [154]. In another similar report, McAuliffe et al. showed that the Notch signaling pathway has a critical regulatory function in ovarian CSCs regulation [155].

**Fig. 3:**
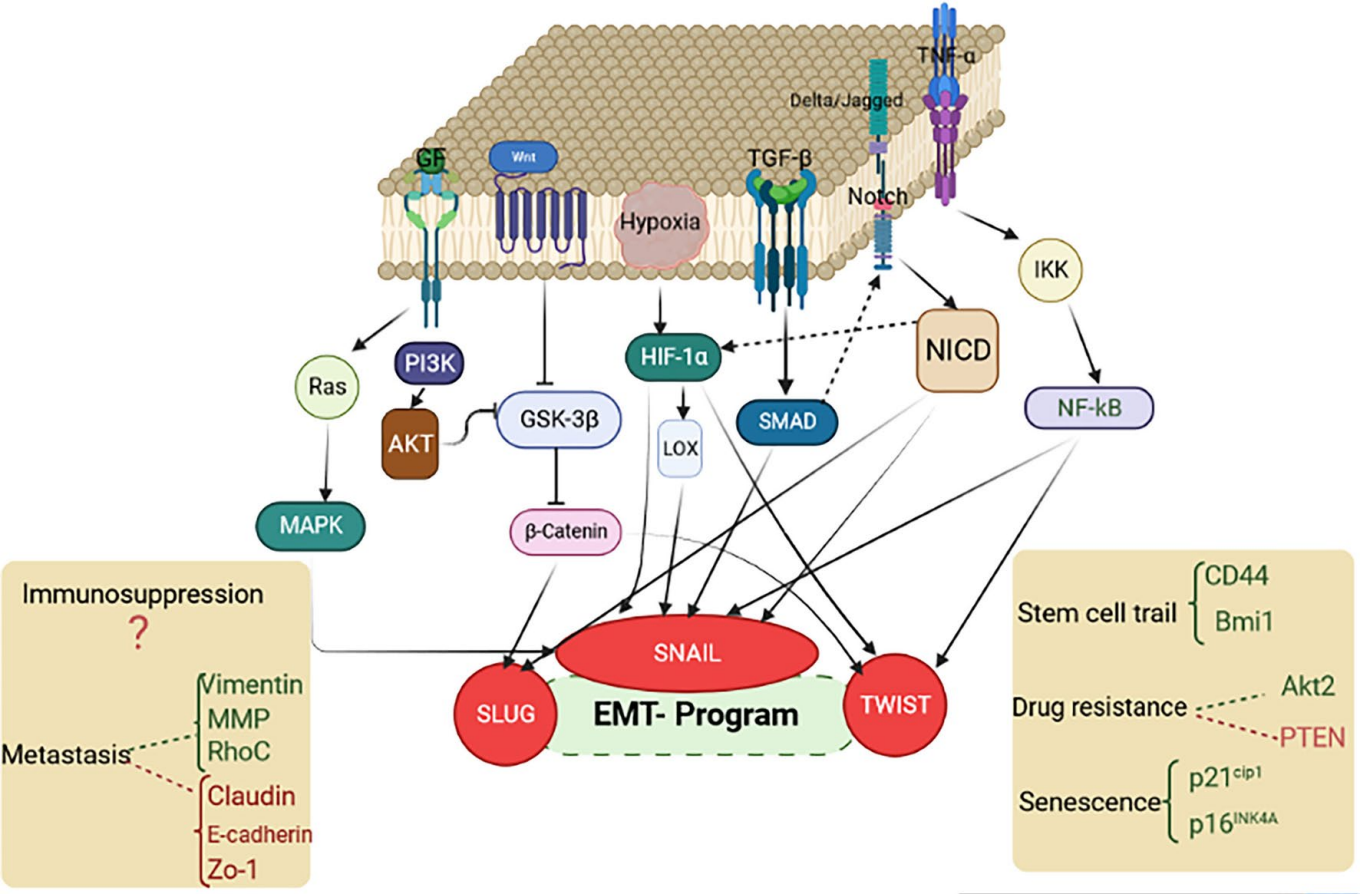
Overview of signaling networks contribute to EMT process and metastatic traits. Activation of Notch, Wnt, tumor growth factor-β (TGF-β), receptor tyrosine kinases (RTKs), and tumor necrosis factor-α (TNF-α) pathways result in the activation of EMT-associated transcription factors (e.g., Snail, Slug, and Twist), that ultimately induce EMT process. As a result of EMT, tumor cells acquire stem cell-like features such as resistance against senescence, immunosuppressive mechanism, chemotherapeutic agents and endocrine therapy in the metastatic cascade (illustration created by biorender.com). This figure adapted from Fig. 1 of reference number 157 [157]

Growing evidence points out that the PI3K/AKT pathway upregulates enzymes involved in ROS scavenging. Alternatively, ROS can promote CSC maintenance by stimulating the Notch signaling pathway. Endothelial cells can release nitric oxide and activate the Notch pathway, which increases stemness in PDGF-triggered gliomas. In this context, Charles et al. provided evidence that the nitric oxide pathway activates notch signaling in human glioma cells, enhancing the side population phenotype [156]. In Fig. 3, we discuss the major signaling pathways that regulate EMT during breast cancer progression and metastasis, specifically focusing on the role of Snail in this complex signaling network [157].

## TFs that regulate the EMT process

It is becoming clear that metabolic reprogramming is a highly regulated process that is mediated by TFs. These regulators are positively correlated with EMT development [6]. Snai1, Snail2, Twist 1, Twist 2, ZEB1, and ZEB2 are the most extensively studied TFs coordinating the EMT process. All of them inhibit E-cadherin expression and support the transition to mesenchymal phenotype. EMT-TFs share common physiological functions during the developing organism and embryogenesis.

**Fig. 4:**
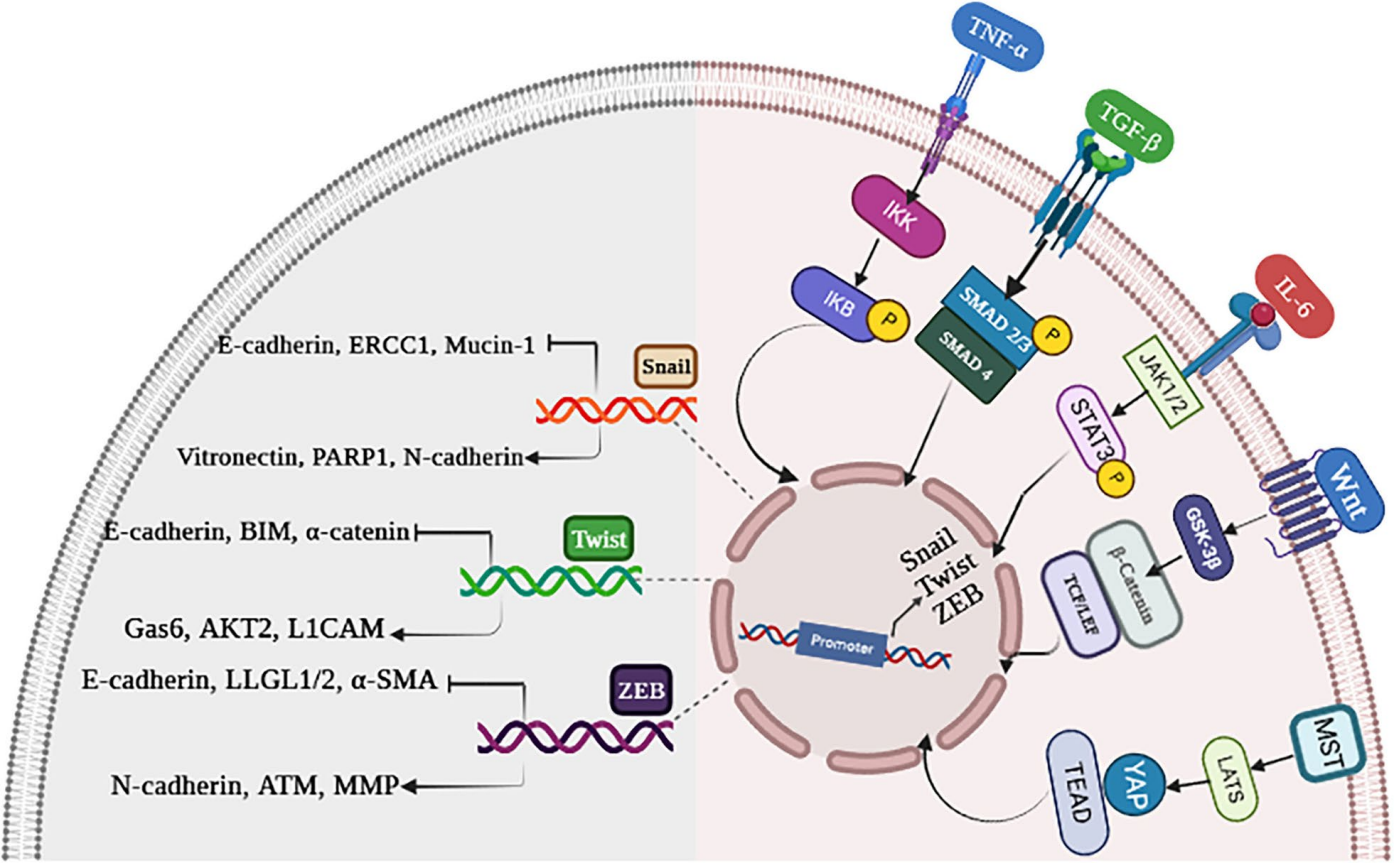
A brief description of key intracellular cascades and transcriptional target genes of EMT-related transcription factors (TFs). **A** EMT-TFs transcription is induced by multiple intracellular pathways via binding to their promoters. **B** EMT-TFs regulate genes that are essential for EMT program along with drug-resistant genes. NF-κB nuclear factor-κB, IKK IκB kinase, TNF-α tumor necrosis factor-α, TNFR tumor necrosis factor receptor, TNF-β transforming growth factor beta, GSK-3β glycogen synthase kinase-3β, TCF/LEF T cell factor/lymphoid enhancer factor, HIF1α hypoxia-inducible factor 1 α, IL-6 interleukin-6, JAK1/2 Janus kinase 1/2, STAT3 signal transducers and activators of transcription 3, PARP1 poly (ADP-ribose) polymerase, ERCC1 excision repair cross-complementing group 1, GAS6 growth arrest-specific 6, L1CAM L1 cell adhesion molecule, BIM Bcl-2-like protein 11, ZO-1 zonula occludens-1, ATM ataxia–telangiectasia-mutated, LLGL1/2 lethal giant larvae protein homolog 1/2, α-SMA α-smooth muscle actin (illustration created by biorender.com). This figure adapted from Fig. 3 of reference number 161 [161]

Interestingly, the reappearance of EMT-TFs in cancer cells is highly correlated with cancer development and progression [158]. Upon activation of EMT-TFs by signaling cascades, transcriptional programs are switched. The clinical relevance of EMT-TFs is found in metastasis, and their expression is associated with poor clinical prognosis in cancer patients (Fig. 4) [159–161].

### Snail1 and Snail2

Snail subfamily members are among the larger family of Zinc finger TFs in humans, composed of Snail1, Snail2 (also known as SLUG), and Snail3. All members act as transcriptional repressors. This family of TFs has a central role in the regulation of EMT. Snail1 and Snail2 are the most widely studied members within this family that trigger the EMT process. There is a strong expression of the Snail family in various cancers, regulating proliferation, survival invasion, and metastasis in cancer cells [162]. Snail proteins have similar structural characteristics. There are four to six C2H2 zinc finger motifs at the carboxyl terminus, which facilitate the direct binding of the protein to DNA. It is known that Snail family proteins bind to DNA using the CAGGTG motif, which is a subset of the E-box sequence so that a variety of basic helix-loop-helix transcription factors can bind to this site. Snail genes contribute to the EMT process in several types of cancers via suppressing the expression of epithelial markers, including E-cadherin, vascular endothelial (VE) cadherin, occluding, claudin, desmoplakin, mucin, and cytokeratin, indicating an epithelial phenotype. In this case, mesenchymal markers such as fibronectin and vitronectin are upregulated, indicating mesenchymal phenotype. In other words, the Snail-induced EMT (Snail1/Snail2) suppresses E-cadherin, preventing cell adhesion and enhancing migration in cancer cells [163, 164]. As a molecular mechanism, Snail2 (Slug) modulates EMT development by transcriptionally repressing the epithelial E-cadherin expression. Indeed, a low Snail transcription and a high E-cadherin expression in epithelial cells can prevent NK-B stimulation and other related signaling pathways from being stimulated in epithelial cells [165]. It has been revealed that TGF-β, as an external stimulus, can activate protein expression of Snail1/Snail2, which ultimately binds to the corresponding Snail genes [166]. Upon suppression of E-cadherin, Snail1 and Snail2 expression levels are amplified, which is mediated by a self-stimulation loop resulting from NF-κB suppression. Therefore, the downregulation of E-cadherin by Snail-mediated repression (Snail1/Snail2) increases the activity of the self-stimulation loop [167]. Moreover, NF-κB activation induces the expression of mesenchymal genes and some repressors, such as ZEB11, inhibiting Snail function by ZEB1-mediated activity with no phenotype reversion. This is why the Snail (Snail1/Snail2) genes are necessary for driving the EMT process. Snail1 and Snail2 are repressing factors of E-cadherin, participating in EMT development [168].

### Twist1 and Twist2

Twist-family plays a pivotal function in a variety of essential developmental processes. Twist proteins repress or activate transcriptional procedures by binding to E-box DNA response elements. These proteins are similar in their structures [169]. Growing evidence declares that twist proteins are decisive drivers of tumorigenesis at an early stage. It was found that Twist TFs are overexpressed during cancer initiation, progression, and metastasis and act as a driver of the EMT process. Besides, overexpression of Twist TFs is highly correlated with worse patient outcomes. Metastatic and invasive tumors are directly associated with increased Twist1 expression, which mediates the loss of key epithelial markers such as E-cadherin. Beyond this, Twist TFs increase the expression of mesenchymal markers (e.g., fibronectin, N-cadherin, and vimentin), thereby reducing cell adhesion and promoting cellular motility.

Moreover, Twist proteins were found to induce the cancer stem cells phenotype [170]. Twist activity can be affected by posttranslational modifications, such as phosphorylation. In this regard, MAP kinase phosphorylates Twist 1 protein, promoting invasiveness and EMT process in BCCs. Activated MAPKs such as p38 and c-Jun N-terminal kinase noticeably phosphorylate residue S68 of Twist 1. This phosphorylation augmented the protein levels of Twist 1 without altering its mRNA levels.

On the contrary, inhibition of MAPK activities can decrease the phosphorylated Twist 1 and its protein levels. Indeed, MAPKs are a potent regulator of Twist 1 via stabilizing it for metastasis [171]. In the same manner, phosphorylation of Twist by AKT is also associated with enhanced invasiveness [172]. On the other hand, the phosphorylation of Twist by the inhibitor of kappa B kinase β (IKKβ) caused its degradation [173].

### ZEB1/2

The ZEB family are transcription factors that consist of ZEB1 and ZEB2 proteins. Both proteins interact with bipartite E-boxes via their zinc finger domains. ZEB1 and ZEB2 proteins drive the EMT process by repressing epithelial markers and activating mesenchymal markers [174]. During physiological conditions, they are primarily expressed in diverse tissues, including the heart, CNS, skeletal muscle, and hematopoietic cells. Both ZEB1 and ZEB2 can partly compensate for each other [175]. However, ZEB1 is mainly found in lymphocytes during the development of T lymphocytes, while ZEB2 appears to be expressed in lymphocytes during the development of B lymphocytes in the spleen. These findings indicate that ZEB1 and ZEB2 have different functions and distinct expression patterns in lymphoid tissues [176]. Interestingly, ZEB TFs may display antagonist functions. It is worth noting that ZEB2 knockout mice die at the embryonic stage; thereby, it can be dedicated that ZEB1 cannot completely compensate for the function of ZEB2 [177]. Several signaling molecules modulate the expression of ZEB1 and ZEB2; for instance, estrogen signaling can stimulate ZEB1 expression. Likewise, it was found that TGFβ and Wnt/β-catenin signaling can activate ZEB1 expression [178, 179]. Furthermore, Snail1 and Twist 1 can cooperatively regulate the expression level of ZEB1 [180]. Further, ZEB1 inhibits the expression of several genes that maintain epithelial cell polarities, such as Lgl2, CDH1, PATJ, and Crumbs3 [181]. From a literature review, it was revealed that Owing to ZEB1/2 expression in epithelial cells, EMT and mesenchymal phenotype are promoted. This results in the invasion, metastatic features, and de-differentiation into cancer stem cells [182]. Chen et al. provided evidence regarding the association of ZEB1expression and poor clinical outcomes in patients with solid tumors, inferring that ZEB1 may be a possible marker to predict prognosis. Noteworthy, cancers that expressed ZEB2 exhibit poor prognosis and survival rates [183].

Together, the loss of E-cadherin function is a crucial hallmark of EMT and invasiveness in diverse types of cancers. Transcriptional repression by Snail1 and Snail2, ZEB1 and ZEB2, and Twist is a key mechanism for the dynamic silencing of CDH1 (the encoding gene of E-cadherin).

## Cancer-associated fibroblasts (CAFs)

Another reason for cancer resistance may be attributed to cancer-associated fibroblasts (CAFs) present in TME of many cancer types. CAFs can affect tumor cells' development, growth, aggressiveness, and metastatic behavior, so targeting CAFs can overcome drug resistance in cancer therapy [184]. As an essential component of the TME, CAFs contribute to cancer growth and progression by triggering extracellular matrix (ECM) deposition and remodeling, exchanging signals with cancer cells, and bidirectional cross talks with immune cells [185]. Accordingly, a better understanding of diverse functions and interactions between normal and tumor fibroblasts, especially CAF subpopulations, can be beneficial in identifying the contributory role of CAFs in cancer development and progression. In this context, determining the communications between cancer and the TME is required to realize how CAFs impact tumor progression and ECM remodeling [186]. In non-malignant conditions such as inflammation or injury, the resident fibroblasts are activated, including the normal activated fibroblasts (NAFs) or fibrosis-associated fibroblasts (FAFs). Notably, the activation of NAFs is similar to CAFs in terms of ECM remodeling and cross-talking with the immune system. However, the mechanisms behind the transition from NAFs to CAFs have not yet been precisely defined. It will be essential to identify the differences and common traits between CAFs and NAFs in the case of cancer or inflammation to know their functions better in the future [187]. In addition, it has been found that tumor-derived CAFs can drive the EMT process in cancers [188]. Vaziri et al. reported that CAFs could use various signaling pathways to promote tumor growth, progression, invasion, and metastatic behavior in some cancers. Based on their findings, CAFs produce leukemia inhibitory factor (LIF) when co-cultured with BCCs. LIF can regulate several important functions, such as cell cycle progression, death, migration, adhesion, and tumorigenesis. Activation of LIF receptor signaling can induce Nanog and Oct4 mechanisms and enhance BCSCs markers CD24^−^/CD44^+^. As a result, targeting LIF and LIF receptor might be a therapeutic approach to breast cancer [189].

## Conclusion

In conclusion, we addressed the current knowledge underlying the EMT process as follows: (a) the contribution of the main metabolic pathways and EMT master transcription factors (e.g., Snail, Twist, and ZEB). In the following, signaling pathways involved in EMT are summarized. Additionally, we discuss how these pathways interact with molecular regulators. As a network of factors and regulators is affected by the metabolic profiles of pre-cancer or cancerous cells. These mechanisms lead to altering cell fate via EMT-mediated modulation, significantly contributing to cancer development. Despite limited information regarding the mechanism by which ROS regulates CSC traits, emerging evidence supports the notion that ROS plays a fundamental role in the self-renewal ability and differentiation capability of CSCs. ROS-dependent signaling networks and related transcriptional programs modulate redox regulation and ROS generation in CSCs. Accordingly, cancer treatment can be improved by targeting CSCs via ROS regulation and antioxidant-related proteins.

## Data Availability

Not applicable.

## Abbreviations

ER: Estrogen receptor
PR: Progesterone receptor
HER2: Human epidermal growth factor receptor 2
TNBC: Triple-negative breast cancer
EMT: Epithelial–mesenchymal transition
MSCs: Mesenchymal stem cells
BCCs: Breast cancer cells
EMT-TFs: EMT-related transcription factors
BCSCs: Breast cancer stem cells
CSCs: Cancer stem cells
TICs: Tumor-initiating cells
ROS: Reactive oxygen species
LPO: Lipid peroxidation
HNE: 4-Hydroxy-2-nonenal
Nrf2/Keap1: Nuclear factor erythroid 2-related factor 2/Kelch-like ECH-associated protein 1
HIF-1α: Hypoxia-inducible factor-1α
ZEB: Zinc finger E-box binding homeobox
TGF-β: Transforming growth factor-β
NF-κB: Nuclear factor kappa B
PI3K: Phosphatidylinositol 3-kinase
ERK: Extracellular signal-regulated kinase
TCA: Tricarboxylic acid cycle
GLS: Glutaminase
FOXC2: Factor Forkhead box C2
GSH: Glutathione synthesis
PKM2: Pyruvate kinase M2
TGIF2: TGF-β-induced factor homeobox-2
HDAC3: Histone deacetylase 3
PKD: Pyruvate dehydrogenase kinase
MET: Mesenchymal–epithelial transition
AMF: Autocrine motility factor
ENO1: Enolase 1
2-PGA: 2-Phosphoglyceric acid
PEP: Phosphoenolpyruvic acid
GLUT: Glucose transporters
MtDNA: Mitochondrial DNA
FASN: Fatty acid synthase
ACC1: Acetyl-CoA carboxylase1
TAK1: Transforming growth factor-activated kinase 1
AKR1B1: Aldo–keto reductase 1 member B1
GD3S: GD3 synthase
·O_2_^−^: Superoxide anion
·NO: Nitric oxide
H_2_O_2_: Hydrogen peroxide
–ONOO: Peroxynitrite
NOXs: NADPH oxidases
NQO1: NAD(P)H: quinone oxidoreductase-1
PK: Pyruvate kinase
AMPK: AMP-activated protein kinase
MMPs: Matrix metalloproteinases
Dlx-2: Distal-less homeobox-2
FPB1: Fructose-1,6-biphosphatase
CtBP: C-terminal binding protein
PIP3: Phosphatidylinositol 3,4,5-trisphosphate
PIP2: Phosphatidylinositol 4,5-bisphosphate
VEGF: Vascular endothelial growth factor
GICs: Glioma-initiating cells
FBP1: Fructose-1,6-bisphosphatase
TCF4: Transcription factor 4
FOXO3a: Forkhead box O3
STAT3: Signal transducer and activator of transcription 3
JAK: Janus kinase signaling pathway
ALDH: Aldehyde dehydrogenase
HIFs: Hypoxia-inducible factors
VE: Vascular endothelial
CAFs: Cancer-associated fibroblasts
ECM: Extracellular matrix
NAFs: Normal activated fibroblasts
LIF: Leukemia inhibitory factor
